# Fungal Genomic Resources for Strain Identification and Diversity Analysis of 1900 Fungal Species

**DOI:** 10.3390/jof7040288

**Published:** 2021-04-12

**Authors:** Mir Asif Iquebal, Sarika Jaiswal, Vineet Kumar Mishra, Rahul Singh Jasrotia, Ulavappa B. Angadi, Bhim Pratap Singh, Ajit Kumar Passari, Purbajyoti Deka, Ratna Prabha, Dhananjaya P. Singh, Vijai Kumar Gupta, Rukam Singh Tomar, Harinder Singh Oberoi, Anil Rai, Dinesh Kumar

**Affiliations:** 1 Centre for Agricultural Bioinformatics, ICAR-Indian Agricultural Statistics Research Institute, Library Avenue, PUSA, New Delhi 110012, India; ma.iquebal@icar.gov.in (M.A.I.); sarika@icar.gov.in (S.J.); rahuljasrotia86@gmail.com (R.S.J.); ub.angadi@icar.gov.in (U.B.A.); anil.rai@icar.gov.in (A.R.); 2 Department of Biotechnology, Mizoram University, Mizoram 796004, India; vineetkumarmishra88@gmail.com (V.K.M.); bhimpratap@gmail.com (B.P.S.); ajit.passari22@gmail.com (A.K.P.); purba562@gmail.com (P.D.); 3 Department of Agriculture & Environmental Sciences, National Institute of Food Technology Entrepreneurship & Management (NIFTEM), Kundli, Sonepat, Haryana 131028, India; 4 ICAR-National Bureau of Agriculturally Important Microorganisms, Kushmaur, Mau Nath Bhanjan 275101, India; ratnasinghbiotech30@gmail.com (R.P.); dhananjaya.singh@icar.gov.in (D.P.S.); 5 Department of Biological Sciences, Sam Higginbottom University of Agriculture, Technology & Sciences, Allahabad 211007, India; vijaifzd@gmail.com; 6 Biorefining and Advanced Materials Research Center, Scotland’s Rural College (SRUC), Kings Buildings, West Mains Road, Edinburgh EH9 3JG, UK; 7 Department of Biochemistry and Biotechnology, Junagadh Agricultural University, Junagadh, Gujarat 362001, India; rukam@jau.in; 8 Food Safety and Standards Authority of India, FDA, Bhavan, Kotla Road, New Delhi 110002, India; harinder.oberoi@icar.gov.in

**Keywords:** fungus, database, FungSatDB, microsatellite, simple sequence repeats (SSRs), genotyping

## Abstract

Identification and diversity analysis of fungi is greatly challenging. Though internal transcribed spacer (ITS), region-based DNA fingerprinting works as a “gold standard” for most of the fungal species group, it cannot differentiate between all the groups and cryptic species. Therefore, it is of paramount importance to find an alternative approach for strain differentiation. Availability of whole genome sequence data of nearly 2000 fungal species are a promising solution to such requirement. We present whole genome sequence-based world’s largest microsatellite database, FungSatDB having >19M loci obtained from >1900 fungal species/strains using >4000 assemblies across globe. Genotyping efficacy of FungSatDB has been evaluated by both in-silico and in-vitro PCR. By in silico PCR, 66 strains of 8 countries representing four continents were successfully differentiated. Genotyping efficacy was also evaluated by in vitro PCR in four fungal species. This approach overcomes limitation of ITS in species, strain signature, and diversity analysis. It can accelerate fungal genomic research endeavors in agriculture, industrial, and environmental management.

## 1. Introduction

Among eukaryotes, fungi represent the second largest group after bacteria, having species ranging from 0.005 million to 5.1 million in numbers over the years [1]. They play significant roles in human life due to their diversity in nutritional and secondary metabolism in natural and artificial niches [2]. Identification of fungal strains has a high relevance in basic knowledge discovery like ecology and taxonomy as well as applied applications in agriculture, industrial, and environmental management. Fungi can be identified by polyphasic approach using their morphological, biochemical, and molecular techniques [3]. In molecular approach, DNA barcode of internal transcribed spacer (ITS) region and proteomics data are more prevalent. Though ITS region amplification using universal primer is accepted as “gold standard” for fungal species identification [4,5], there are approximately 30% of fungal species where identification may not work beyond genus level, such as in (many species of lineage *Pezizomycotina*, *Morerellomycotina*, *Kickxellomycotina*, *Zoopagomycotina*, *Mucoromycotina*, *Entomophthoromycotina*, *Chytridiomycotina*, *Neocallimasgomycotina*, and *Blastocladiomycotina* [5,6]. Such limitations also exist in differentiation of cryptic species [7]. There is a noticeable gap in sub- species or strain level of ITS region barcode of fungal species signature. 

Though proteomics-based approaches are effective for differentiation at species and strain levels, they are neither rapid nor economical. Moreover, such data management in the form of molecular voucher specimen (like simple sequence repeats (SSR) profile/allele signature) and universalization of methodology for across lab/country will pose further challenges [8]. Since ITS based methods use GenBank Blast search which is dependent on existing fungal sequences that are often improperly described. This problem is further compounded by the rapidly changing nature of fungal taxonomy and lack of strain level data [7]. 

Genotyping based mycological diversity and identification face major global challenges, such as non-availability of simple, cost effective, and rapid identification, user friendly molecular data, ability to trace origin and biogeography of the strains [9]. In such challenging situations, different molecular approaches, like RAPD, MLSA, MLSD, SNP, CGH, and MALDI-TOF MS can be used to resolve strain level differentiation. Among these, SSR markers are better choice because of their high reproducibility, greater discriminatory power, amenability to multiplexing and relatively more cost effective for screening large number of samples [9]. Thus, use of SSRs can be promising in diversity and identification of fungi strains along with population structure and genetic relatedness due to the presence of higher number of alleles [10]. 

Microsatellites or simple sequence repeats (SSRs) are short, hypervariable, tandemly repeats (1–6 bp), generated by slippage during DNA replication and thus have higher degree of polymorphism. Being highly variable codominant markers and neutral in selection, following Mendelian inheritance, it is a marker of choice for population structure, differentiation, and population assignment [11].

Fungal SSR profile has been found capable to be used as DNA signature for species, sub-species, strains, and sub-strains/isolate, such as strain differentiation between harmful and beneficial *Aspergilli* [12]. Allele specific fungal species identification is also reported, for example, poplar rust fungal species differentiation, *Melampsoma medusa* and *M. larici-populina* [13,14]. This demonstrates that single or multi locus genotyping of SSR loci can be used to obtain species, subspecies, or strain specific DNA signature [15].

SSR based microbial identification has a significant advantage as they can be used without having the need to culture fungi, thereby making the process rapid by direct sample analysis [6]. SSR based approach can give much higher specificity and sensitivity in fungal diagnostic tests even at significantly lower titers, which cannot be achieved by morphological and biochemical approaches [16]. Before availability of whole genome sequence discovery of fungal SSRs were done by developing genomic library and sequencing of repeat bearing clones. This was time-consuming and very expensive. The use of such markers in heterologous species were limited in number due to lower cross amplifiability, lower number of alleles, higher homozygosity and null alleles [17]. With the advent of Next Generation Sequencing (NGS) technology, which is becoming cheaper day by day, in silico mining of SSR offers a great advantage over genomic library based methods in terms of cost and rapidity [18]. Previously, whole genome based fungal species microsatellite genome wide survey reports were limited to either a few species or without any database. For example, FungREP 1.0 is having limited 44 fungal species [19]. In another study, where SSR survey has been made in nine fungal species genomes but it is without any genomic resource [20].

As on today, there are >1900 fungal species with their WGS represented by >4000 assemblies. The number of fungal species is rapidly increasing across globe, thus, there is a need to develop a single web-genomic resource of all available fungal genomes with genome wide SSR mining, thus paving the way for universalized approach for genotyping, catering to the need of identification, differentiation, and management of fungal germplasm diversity by global community. It can facilitate pan-global community in implementing the Convention of Biological Diversity (CBD) and the Nagoya Protocol (NP), where both depositors and users may require addressing the issues of identity, traceability, and sovereignty in access benefit sharing (ABS) of fungal germplasm [21], especially in dispute/violation of material transfer agreement (MTA). Thus, there is a need to develop a web genomic resource having all available fungal genome and its genome wide SSRs mining with ready to use primers for genotyping. Such resource can accelerate the fungal diversity analysis research and management of fungal germplasm by global community.

Various SSR databases and information are available for fungal genome in literature but these are limited to few species. For example, SSR survey of nine fungal species [20], Fungrep 1.0 with 44 fungal species [19], SSRome, which is a comprehensive database of 6533 organisms housing 241 fungal genomes [22], species specific microsatellite database of *Saccharomyces cerevisiae* [23] and group specific pathogenic fungi [24]. Fungal MLST database, an International Fungal Multi Locus sequence typing database developed in consortium for 10 fungal species (https://mlst.mycologylab.org/ (accessed on 15 October 2020)) and FungiDB, a Fungal and Oomycete Information Resources (https://fungidb.org/fungidb/app (accessed on 15 October 2020)) also have the information of fungal SSRs of 164 species. An extensive SSR database of fungal genome, MSDB populated with 46,122 eukaryotic species which includes 5804 fungal species genomes [25]. However, there is no comprehensive, single fungal specific database covering whole genome based SSR loci with location specific primers for rapid genotyping required for diversity analysis and strain differentiation. Genotyping of selected SSR loci with genomic coordinates enables the user to have optimal set of markers to represent variability within the genome and population of a species gene pool. 

To the best of our knowledge, we present here the world’s first whole genome sequence-based microsatellite database having ready to use primers for multi-locus genotyping of >1900 fungal species based on the analysis of >4000 genomes covering major three classes of fungi for population diversity structure and differentiation of species.

## 2. Materials and Methods

### 2.1. Fungal Genomic Data Source 

A total of 1973 species were selected from NCBI Genome Database having more than 5000 fungal genome assemblies (http://www.ncbi.nlm.nih.gov/genome/ (accessed on 15 October 2020)). Fungal assemblies were selected based on the criteria of availability of whole genome assembly, redundancy of species and genome finishing. Out of 1973 species, 1410 belonged to Ascomycetes, followed by 430 and 133 species to Basidiomycetes and other fungi classes, respectively. Various parameters like sub-group, size, GC content, number of genes and protein data of genome assembly of each species and strains/isolates were also collected.

### 2.2. Genome Wide SSR Mining and Primer Designing for Genotyping 

Pre-processing of genomic data was performed using perl script of est_trimmer.pl [26]. Perl script of MISA (MIcroSAtellite identification tool) tool was used for the genome-wide SSR mining with default parameters i.e., 10 repeating units for mononucleotides, six repeating units for dinucleotides and five repeating units for trinucleotides, tetranucleotides, pentanucleotides and hexanucleotides [27]. In order to get complex and compound markers, modifications were done in the misa perl script. Descriptive information of the mined markers, such as repeat numbers, marker type, GC content, markers size, start and end location were obtained. Further, Primer 3 core executable was integrated in the fungal SSR database for the generation of primer pairs of each locus for PCR based genotyping [28]. Parameters used for the primer generation were: primer size 18–27 bp length, 55–65 °C melting temperature, product size ranging between 150–280 bp, and GC content of 40–70%. 

### 2.3. Development of Web Genomic Resource: FungSatDB

**Figure 1:**
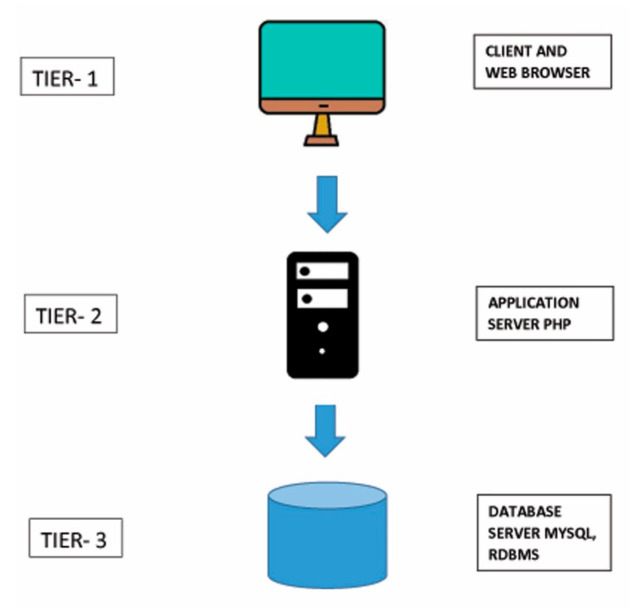
Three-tier architecture of FungSatDB.

FungSatDB based on 3-tier architecture was designed for developing microsatellites in fungal genomes using LAMP (Linux-Apache-MySQL-PHP) technology (Figure 1). Provision was also made to furnish the information about the primers designed across flanking region of SSR loci. This was done to facilitate the users for cost and time effective genotyping, so that a user can design new primer at desired location in the genome as well as use known published primer (external primers) in ePCR mode in the selected assembly. This application has PERL script embedded at the backend which computes the amplicon size in terms of basepairs by aligning forward and reverse primer over template genome. Provision is also made for inclusion of new fungal genome assembly for future updates as and when the reference sequence is available.

### 2.4. In Silico PCR Based Evaluation of Strain Differentiation Ability of FungSatDB

To evaluate the efficacy of our database for strain differentiation, we selected a widely present fungal species *Fusarium oxysporum.* A total of 66 genome assemblies (https://www.ncbi.nlm.nih.gov/genome/genomes/707 (accessed on 15 October 2020)) of different isolates of this species from four different continents (Asia, Australia/Oceania, Europe, North America) were selected for strain differentiation by SSR polymorphism using e-PCR. In silico genotyping of 75 SSR loci was carried out using ePCR of online tool [29]. Five simple and di- nucleotide repeat loci were selected from each of the 15 chromosomes which are expected to have a high degree of polymorphism and thus better representation of variability of both genome as well as population. Multilocus allelic data was generated using e-PCR option. In order to evaluate effectiveness of allelic profile of these strains, hierarchical clustering using average distance-based method was performed. This cluster analysis was done using codes in R language.

### 2.5. In Vitro PCR Genotyping Evaluation of SSRs from FungSatDB

In order to evaluate widest applicability of FungSatDB, four divergent classes, i.e., Eurotiomycetes (species *Aspergillus flavus*), Saccharomycetes (species *Candida albicans*), Dothideomycetes (species *Macrophomina phaseolorum*), and Sordariomycetes (species *Trichoderma longibrachiatum*), where one species each were selected. In order to ensure species identity critically required for such work, isolates for first three fungal species were taken from International Depository Authority (IDA) recognized repository in India. Aspergillus flavus: MTCC 9064 and Candida albicans: MTCC 3017 were obtained from IMTECH, Chandigarh, India. Mirohina fasionima: NAIMCC-F-01260 was obtained from ICAR-NBAIM, Mau, UP, India. Species identity of *Trichoderma longibrachiatum* AC2 was ensured by standard fungal species barcoding of ITS region sequences. 

### 2.6. Identification of Trichoderma Longibrachiatum Using ITS rRNA Sequencing

Fungal endophyte *Trichoderma longibrachiatum* isolate AC2 was isolated from leaves of *Anthocephalus cadamba* (Roxb.) Miq. The total genomic DNA was isolated from pure mycelia by following the procedure reported by [30] Cenis, (1992). The DNA was then subjected to PCR amplification of ITS region (ITS1-5.8S-ITS2) using universal primers ITS1 (5′-TCCGTAGGTGAACCTGCGG-3′) and ITS4 (5′-TCCTCCGCTTATTGATATGC-3′) [31] using Veriti thermal cycler (Applied Biosystems, Singapore, Republic of Singapore). The 25 µL PCR reaction consists of 1× PCR assay buffer, 1.5 mM MgCl_2_, 200 µm of each dNTPs, 10 pmols of each primer, 50 ng template DNA, and 1 U Taq DNA polymerase. The PCR conditions were as follows: 95 °C for 5 min; with 35 cycles of denaturation for 1 min. at 95 °C, annealing at 55 °C for 1 min, extension at 72 °C for 1 min 20 sec; and 72 °C for 10 min. The amplified PCR products (2 μL) were visualized on 1.5% (*w*/*v*) agarose gel prepared in 1× TBE buffer by using gel documentation system (Bio-Rad Gel Doc XR+ gel documentation system, Hercules, California City, CA, USA). Sequencing was done commercially at Sci Genome Pvt. Ltd. Kochin, India. The chromatograms of the obtained sequences were analyzed using Finch TV v1.40v. The sequence having high level of sequence similarity (97–100%) obtained by using BLASTn search was considered as closest match. The nucleotide sequence of ITS rDNA was submitted to NCBI GenBank and accession number KX655582 of the isolate was obtained.

### 2.7. In Vitro PCR Validation

ePCR amplicons obtained over 10 loci were compared with each of the four species using the same set of primers. Genomic DNA of each fungal strain was isolated using protocol as described by [32]. Species identities of these fungal isolates were already confirmed by ITS1 and ITS4 region sequencing followed by BLAST analysis [31]. Primer3 was used for primer designing of 10 loci in each of the for species using accession number (*Aspergillus flavus* (GCA_000006275.2), *Candida albicans* (GCA_000784595.1), *Macrophomina phaseolorum* (GCA_000302655.1), and *Trichoderma longibrachiatum* (GCA_000332775.1)). Primer sequences are furnished in Supplementary File S1. The PCR reactions were carried out in Verity thermal cycler (Applied Biosystems, Singapore). Each reaction contained 50 ng of genomic DNA, 1× PCR assay buffer, 1.5–2.5 mM MgCl_2_ (varied concentration for different set of reaction mixture), 2.5 mm dNTPs, 10 pmols of each primer and 1.5 unit of Taq DNA polymerase. The thermal cycler conditions were: initial denaturation at 95 °C for 5 min, followed by 30 cycles of denaturation at 94 °C for 1 min, annealing (52–59 °C) for 1 min, and extension at 72 °C for 2 min and a final extension at 72 °C for 10 min.

## 3. Results and Discussion

### 3.1. Genome Wide SSR Mining and Primer Designing for Genotyping 

**Figure 2:**
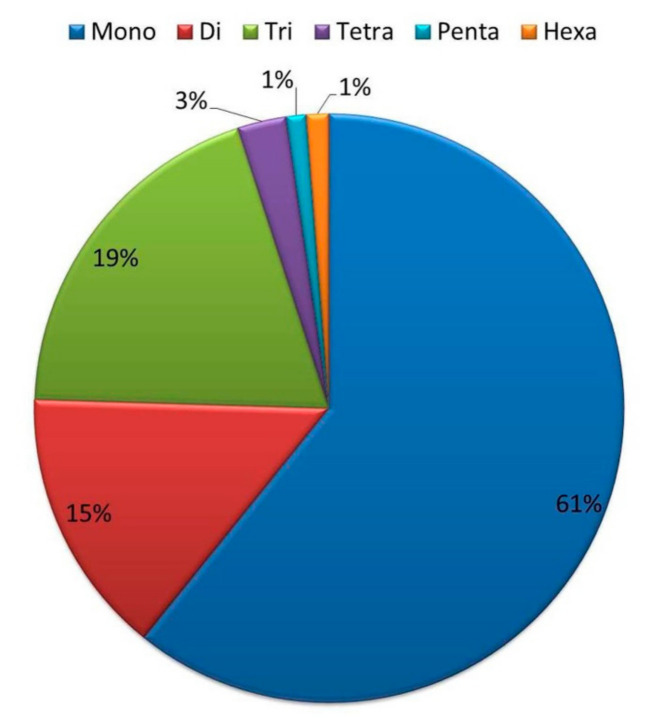
Percentage distribution of the simple sequence repeats (SSR) motif type.

**Figure 3:**
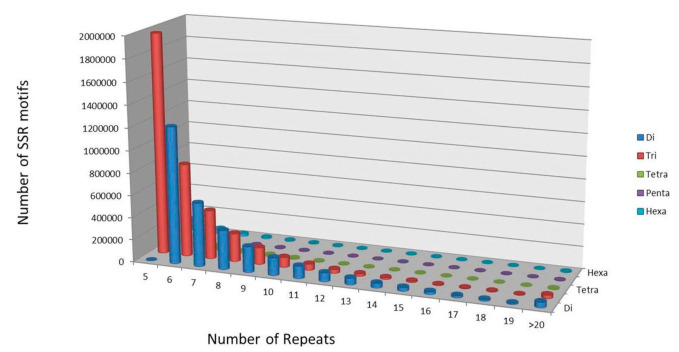
Motif and repeat abundance in fungal whole genome based SSR survey.

Out of the available >5000 available fungal genome sequences, partially finished genome sequences along with species redundancy were excluded. SSR markers were successfully mined from all the remaining 3903 genome assemblies. A total of 19,079,777 markers were mined from these genome assemblies. Maximum repeats were found in mononucleotide i.e., 11,602,011 repeats (61%), followed by 3,723,047 (19%), 2,798,128 (16%), 513,900 (3%), 235,028 (1%), and 207,663 (1%), repeats in trinucleotides, dinucleotides, tetranucleotides, hexanucleotides, and pentanucleotides, respectively (Figure 2). Our all-fungal genome SSRs survey reveals that mono-, di-, and tri-nucleotide repeats were higher in abundance with respect to longer repeats (Figure 3) (Karaoglu et al., 2004). We found the second highest abundance of tri-nucleotide repeats (19%) which is due to exonic motifs [33]. This fungal genome’s survey also reveals huge size differences of thousand folds (from 2.2 Mb to 2.1 GB) but SSR distribution, abundance, and density is independent of respective genome sizes [20].

Our database offers advantage to users before SSR validation by in vitro PCR as all duplicate loci present in the genome can be removed. Duplicates giving multiple bands can be avoided [34]. This approach can reduce the probability of multiple bands drastically, which is often in the range of 20–30%. Moreover, now cost of SSR genotyping for allelic length polymorphism using multiplexing with fluorescent dyes has become costly affair with respect to sequencing of the amplicon. SSR length polymorphism has been found most economical in diversity studies but such data is devoid of magnitude of structural polymorphism over these loci. Our database can be used for both, structural as well as length polymorphism thereby offering relatively higher sensitivity in strain differentiation. Such structural polymorphism of SSR can be used to burst “null alleles” especially encountered in heterologous mode of SSR use when genome sequence of the fungi is not available.

### 3.2. Development of Web Genomic Resource: FungSatDB

**Figure 4:**
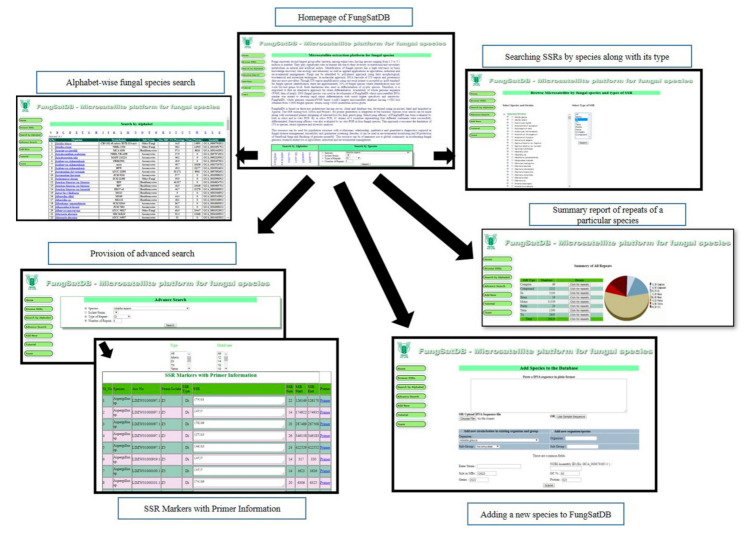
Flow chart of FungSatDB which includes mining pipeline of markers identification, analysis, structure, and development.

FungSatDB, freely accessible at http://webtom.cabgrid.res.in/fungsatdb/ (accessed on 22 March 2020) is an exclusive database of microsatellite repeats for various fungal genome cataloguing information of 19,079,777 repeats which contains 10,235,086 simple, 1,459,190 compound, and 56484 complex markers from 3903 assemblies of 1973 species. User can obtain different types and motifs of microsatellites (simple, compound, and complex), along with their location and length in the genome assembly of these fungal species. The web interface of FungSatDB includes concise information about the database and links to the different pages from where information about different class of microsatellite markers can be accessed. FungSatDB has four separate tabs, namely, “Home”, “Microsatellites”, “Analysis”, and “Team” that offers common information about the database, details of the species included, approach used for in silico microsatellite mining and primer designing and the team involved in development, (Figure 4). This database provides two different ways for marker search such as “Species search” and “Alphabetic search”. Microsatellites for any species can be accessed directly by selecting the species name in “Species search” option, followed by selecting various parameters, such as isolate, types of repeats and number of repeats. Such repeat selection has an advantage in getting potentially highly polymorphic loci as simple and longer repeats generate a greater number of alleles due to higher mutation rate of such loci [35]. The user can view the searched markers results with bar graph for marker distribution. For each species, a separate page is provided that includes details of total repeats identified along with total number of microsatellites of perfect (mono- to hexa-nucleotide), compound and complex type. In “*Details*” column, hyperlink is provided at “*Click for repeats*” to generate dynamic page having entire repeats of that particular category. The user can select any repeat loci to obtain its primer with parameters of their choice. Primer sequence is displayed with its full information on forward and reverse sequence, amplicon size, melting temperature, GC content, start position, and product size. 

### 3.3. In Silico PCR Based Evaluation of Strain Differentiation Ability of FungSatDB

**Figure 5:**
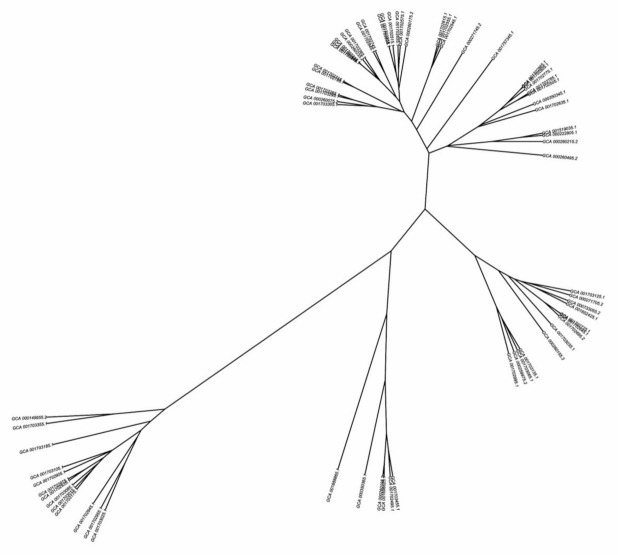
Effective differentiation of 66 strains of *F. oxysporum* by clustering of ePCR alleles of 75 loci.

Successful differentiation of all selected 66 isolates of *F. oxysporum* was obtained by ePCR using primers generated over 5 loci on each of the 15 different chromosomes. Strain-wise detailed allelic profiles of all these 75 loci are available in Supplementary file (Supplementary File S2). Generated allelic data is also presented in pictorial form as cluster analysis using R scripts. Since selected isolates were from four continents (Asia, Australia/Oceania, Europe, North America) representing eight countries (India, China, Malaysia, Japan, Australia, Netherlands, Switzerland, USA) it clearly demonstrates the immense utility of FungSatDB to differentiate all the strains of fungi sequenced so far across globe (Figure 5). In a given fungal species, if allelic data are obtained from each chromosome with an equal number of loci, such data represents variability profile with homogeneity within its genome.

Our in silico strain differentiation also demonstrates that all the 75 loci are not necessary for strain differentiation. It is expected that genome assembly of different strains may vary in terms of size, thus missing loci cannot be ruled out. For example, GCA_001703185.1 and GCA_001703175.1 have 21 and 11 missing loci but still they are well differentiated. It is interesting to note that assembly GCA_001888865.1 having 65 missing loci also got differentiated.

Though this differentiation of fungal strains by SSR loci has been reported in large number of species of both, ascomycetes [36] and basidiomycetes [37], most of them by in vitro PCR with limited number of markers. This genomic resource offers two major advantages to fungal research community: (1) SSR mining covers entire genome of a given species in rapid and economical mode with respect to SSR discovery by genomic library method, (2) potential polymorphic loci can easily be selected by using tools of ePCR which can differentiate strains with minimum number. 

### 3.4. In Vitro PCR Genotyping Evaluation of SSRs from FungSatDB

**Figure 6:**
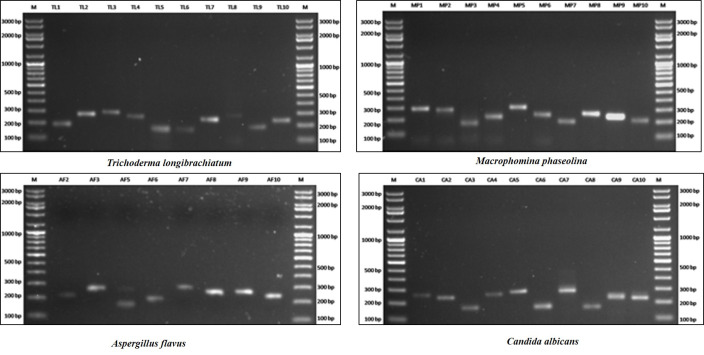
In vitro PCR genotyping evaluation of SSRs from FungSatDB.

In silico SSR mining approach and chromosome-wise optimal set of SSR loci selection based on genomic coordinates can be advantageous in terms of time and cost required for genotyping based strain differentiation before in vitro PCR. In silico approach has the additional advantage of getting polymorphic loci enlisted if more than one genome assembly is available along with selection of type of repeat and location in the genome. The degree of polymorphism and allelic length difference were found in each of the 75 loci represented by 5 loci on each of the 15 different chromosomes (File S2). In vitro PCR amplifications were successful for 38 out of 40 (95%) of the loci, i.e., 10 loci for four species each (Figure 6). It clearly demonstrates the successful use of FungSatDB in genotyping. This clearly reveals that every isolate of this species can easily be differentiated. This approach overcomes on the limitation [38] of ITS based species bar-code for strain differentiation.

SSR primers designed on genome assembly of fungal species *Fusarium oxysporum* were used for ePCR of 66 genome assemblies of different strains as template (https://www.ncbi.nlm.nih.gov/genome/genomes/707 (accessed on 15 October 2020)) pertaining to four different continents and eight countries. Allelic polymorphic data of SSR loci were used for clustering to evaluate their effectiveness for strain differentiation. While selecting the locus for allelic data generation using ePCR, locus selection was done on following parameters: Five loci per chromosome, simple repeat, longer repeat length, flanking regions not having repeats as primers cannot be designed with higher specificity in unique regions only.

### 3.5. Utility of FungSatDB

The major utility of FungSatDB is to overcome the two main limitations of “gold standard” method of ITS region fingerprinting in fungal species and strain differentiation. There are at least 30% fungal species which cannot be differentiated by ITS region fingerprinting creating a species barcode gap [6,7]. Moreover, such a gap is absolute in sub species/strains [5] as well as also in cryptic species differentiation [39]. Present fungal genomic resource can be used to address all such major challenges in fungal diversity analysis and identification. Molecular level fungal identification methods are much more accurate due to their sensitivity and specificity [40]. It can work even without culturing the fungi. The developed genomic resource can accelerate rapid, specific, sensitive, and cost-effective use in major areas of agricultural, industrial, and medical sectors. 

Present genomic resources can resolve the issue of economically important cryptic fungal strains differentiation. For example, SSR based differentiation of *Periconia epilithographicola* and *Coniochaeta cipronana* fungi having higher cellulolytic activity for biodegradation ability which is difficult to differentiate using ITS and housekeeping genes signature [41]. In plant fungal pathogen diagnostics where species specific “private alleles” of SSR loci are present has been very effective, for example *Plasmopara viticola* in powdery mildew disease in grape [42], *Macrophomina phaseolina* causing charcoal root rot in cotton and soybean [43], poplar rust fungi (*Melampsoma medusa* and *M. larici-populina*) [13,14]. A combination of public and private allelic richness can make this approach further robust for differentiation of such fungal species, for example differentiation of citrus pathogenic fungal species, *Colletotrichum gloeosporioides* [44]. 

A challenging case of wheat fungal rust pathotype strain differentiation of *Puccinia graminis* f. sp. tritici (Pgt) by SSR has been successfully reported. In this case both strains (TTKSP and TTKS) of Ug99 lineage were phenotypically identical [45] but had a difference in their virulence. In this case, allelic data of SSR loci was pooled with SNPs data very effectively to make lucid differentiation among sub-strains with machine learning approach [46]. Another interesting case of fungal traceability across border has been reported by use of SSR markers differentiating two strains of *Puccinia graminis*, Ug99 and UVPgt55 having South African and North American origin, respectively [45]. Such traceability of fungal strain origin has also been successfully reported in scab disease of apple caused by *Venturia inaequalis* [47]. 

The success of efficient SSR based monitoring of stripe rust pathogen using only 9 loci in wheat in Australia clearly demonstrates that rapid and efficient pathogen identification with minimum risk and time constraints associated with screening of exotic isolates [48] in quarantine centers at port of entry. Such screening has to be robust, reproducible, even with limited DNA quantities. This approach overcomes the limitation of traditional survey work where mating type and fungicide resistance-based studies are not enough to differentiate the isolates along with its center of origin [49]. 

SSR profiling has been found as very successful in clustering of diverging populations depicting its linkages along with pathways, driving evolution and dissemination of pathogen at local as well as global scales. Such information is critically required in developing disease combating strategies where resistant cultivar is selected against specific fungal pathotype which is differentiated by SSR profiling. Thus, it has immense use in optimization of management practices required in crop and pathogen management. In case of fungal outbreak in agricultural crops, such approach can save time and cost required for evaluation of host crop resistance against specific fungal pathotype by rapid SSR fungal genotype profiling. For example, intra and inter-lineage diversity estimation and establishment of global lineage to manage late blight of potato disease caused by different isolates of *Phytopthora* *infestans* [50]. Population genetic structure based on SSR revealing their centre of origin, flow or tracking of host depicting its history and evolutionary potential, as it has been reported in apple scab disease caused by fungi, *Venturia inaequalis* [47]. Within a given fungal pathogenic species, population structure and diversity require a molecular approach. Such studies using SSRs can elucidate the population dynamics of “Shifty enemies” which is critically required in development of pathogen combating strategies [51]. 

Among the best use of beneficial fungi in agriculture, fungal SSRs can be used to measure colonization efficiency of different strains of *Trichoderma virens* as root-endophytic fungi by qPCR. Such approach in agriculture has the advantage in use of fungus as biological fertilizer minimizing chemical fertilizers and pesticides by selecting efficient strains [52]. Another beneficial use of fungal SSR profiling is quantification of fungal load in the soil sample by culture independent method. For example, quantification of two entomopathogenic fungi, *Beauveria bassiana* and *B. brongniartii* in soil samples in maize field which are used for biological control of European cockchafer (*Melolontha melolontha*). Such culture independent approach by direct amplification of fungal SSR loci has several advantages like low cost, rapidity, specificity, sensitivity, and its traceability [53]. Another example of such SSR based differentiation of entomopathogenic fungus *Paecilomyces fumosoroseus* biotype in management of white fly insect transmitted begomovirus disease [54].

Fungal SSR allelic data can be extremely helpful in environmental pollution management where lichens are used as bio-indicators. Perturbation in environment can be deduced to quantify magnitude of it in terms of habitat/ecological fragmentation with pollutant levels. For example, use of lichenized fungal species *Usnea subfloridana* as bioindicator for forest and environmental pollution [55]. Fungal SSR polymorphism has been used in association studies of fungal traits like host specificity, growth rate, and copper resistance [56].

Apart from agricultural and environmental applications, fungal SSR allelic data has immense use in industries like wineries, breweries, and distilleries. For example, critical identification at strain level especially in high commercial value fungal germplasm like ascomycetes, such as *Saccharomyces cerevisiae* used in such industries [57]. It is also promising in establishing the relatedness of industrially important fungi [58]. Efficient fungal strains like *Agaricus*, *Aspergillus*, *Rhizopus*, and *Trichoderma* ssp. used in valorization of industrial and agri-waste and biorefinery [59] can be differentiated using SSR allelic data. Fungal strain differentiation with population structure is required to identify virulent isolates having ability to persist over a long period of time. Such information is valuable as strategic input in the hospital management for control of *Aspergillus fumigatus* causing invasive aspergillosis (IA) [60].

For genome finishing, this largest genomic resource of fungal SSR can be of immense use. Around one-fifth fungal genomes are yet to have genome finishing. It can be used in both HAPPY mapping and optical mapping approach of genome finishing. There is rapid increase in availability of fungal genome data due to low-cost sequencing technology but genome assembly and finishing is a major challenge. Such approach has advantage as it does not involve cloning but covers genomic regions which are even not present in WGS libraries [61], further saving huge cost and time. We believe that FungSatDB having highest number of both, fungal species and whole genome based SSR markers at one place can be of immense use by global community. Such an approach has not only the advantage of species-wise designing of multiplex PCR genotyping but high abundance of SSR also offers an advantage of thermodynamic designing flexibility in selection of locus. Further, there is a pan-global need in terms of traceability and monitoring of exotic fungal strain dissemination especially associated with trans-border trades. 

## 4. Conclusions

Being the world’s largest fungal SSR database, having more than 19 million markers of >1.9 K fungal species, FungSatDB has highly diverse applicability in various sectors of agricultural, industrial, medical, and environmental management. It can not only be used in diversity analyses, but also for DNA signature of isolates/strains. It can be used to study population structure, constructing pedigree, evolutionary relationship, qualitative and quantitative diagnostics for better fungal disease combating strategies like traceability of fungus for its origin, quarantine screening of plant pathogen and population monitoring in environmental management. Further, it can be used in technology management by IP protection of beneficial fungi, survey and management of medical infrastructure and finishing of genome assembly. FungSatDB can be a promising tool for research and services, both, wherever strain identification is of paramount importance. 

## Data Availability

FungSatDB is online freely available and easily accessible from http://webtom.cabgrid.res.in/fungsatdb/ (accessed on 22 March 2020).

## Supplementary Materials

The following are available online at https://www.mdpi.com/article/10.3390/jof7040288/s1, Supplementary File S1. Primer sequences for in vitro PCR genotyping evaluation of SSRs. Supplementary File S2. Strain-wise detailed allelic profile of 75 loci.

## References

[1] Wu B., Hussain M., Zhang W., Stadler M., Liu X., Xiang M. (2019). Current Insights into Fungal Species Diversity and Perspective on Naming the Environmental DNA Sequences of Fungi. Mycology.

[2] Stajich J.E., Berbee M.L., Blackwell M., Hibbett D.S., James T.Y., Spatafora J.W., Taylor J.W. (2009). Primer-The Fungi. Curr. Biol..

[3] Wicht B., Petrini O., Jermini M., Gessler C., Broggini G.A.L. (2012). Molecular, Proteomic and Morphological Characterization of the Ascomycete Guignardia Bidwellii, Agent of Grape Black Rot: A Polyphasic Approach to Fungal Identification. Mycologia.

[4] Martin K.J., Rygiewicz P.T. (2005). Fungal-Specific PCR Primers Developed for Analysis of the ITS Region of Environmental DNA extracts. BMC Microbiol..

[5] Schoch C.L., Seifert K.A., Huhndorf S., Robert V., Spouge J.L., Levesque C.A., Chen W. (2012). Fungal Barcoding Consortium. Nuclear Ribosomal Internal Transcribed Spacer (ITS) Region as a Universal DNA Barcode Marker for Fungi. In Fungal Barcoding Consortium. Proc. Natl. Acad. Sci. USA.

[6] Sun X., Guo L.D. (2012). Endophytic Fungal Diversity: Review of Traditional and Molecular Techniques. Mycology.

[7] Raja H.A., Miller A.N., Pearce C.J., Oberlies N.H. (2017). Fungal Identification Using Molecular Tools: A Primer for the Natural Products Research Community. J. Nat. Prod..

[8] Chikkerur J., Samanta A.K., Dhali A., Kolte A.P., Roy S., Maria P. (2018). In Silico Evaluation and Identification of Fungi Capable of Producing Endo-Inulinase Enzyme. PLoS ONE.

[9] Araujo R. (2014). Towards the Genotyping of Fungi: Methods, Benefits and Challenges. Curr. Fungal Infect. Rep..

[10] Tsykun T., Rellstab C., Dutech C., Sipos G., Prospero S. (2017). Comparative Assessment of SSR and SNP Markers for Inferring the Population Genetic Structure of the Common Fungus Armillaria Cepistipes. Heredity.

[11] Temnykh S., DeClerck G., Lukashova A., Lipovich L., Cartinhour S., McCouch S. (2001). Computational and Experimental Analysis of Microsatellites in Rice (*Oryza Sativa* L.): Frequency, Length Variation, Transposon Associations, and Genetic Marker Potential. Genome Res..

[12] Mahfooz S., Singh S.P., Mishra N., Mishra A. (2017). A Comparison of Microsatellites in Phytopathogenic Aspergillus Species in Order to Develop Markers for the Assessment of Genetic Diversity Among its Isolates. Front. Microbiol..

[13] Steimel J., Chen W., Harrington T.C. (2005). Development and Characterization of Microsatellite Markers for the Poplar Rust Fungi Melampsora Medusae and Melampsora Larici-Populina. Mol. Ecol. Notes.

[14] Galović V., Orlović S., Pap P., Kovačević B., Marković M. (2010). Specificity of SSR Loci for Melampsora Species on Poplars. Genetika.

[15] O’Donnell K., Ward T.J., Aberra D., Kistler H.C., Aoki T., Orwig N., Kimura M., Bjørnstad Å., Klemsdal S.S. (2008). Multilocus Genotyping and Molecular Phylogenetics Resolve a Novel Head Blight Pathogen Within the Fusarium Graminearum Species Complex from Ethiopia. Fungal Genet. Biol..

[16] Stahl P.D., Parkin T.B. (1996). Relationship of Soil Ergosterol Concentration and Fungal Biomass. Soil Biol. Biochem..

[17] Dutech C., Enjalbert J., Fournier E., Delmotte F., Barres B., Carlier J., Tharreau D., Giraud T. (2007). Challenges of Microsatellite Isolation in Fungi. Fungal Genet. Biol..

[18] Vieira M.L.C., Santini L., Diniz A.L., Munhoz C.D.F. (2016). Microsatellite Markers: What They Mean and Why They are so Useful. Genet. Mol. Biol..

[19] Mudunuri S.B., Prabha R., Singh D.P., Krishna G. (2016). FungREP 1.0: Online Web-Repository of Microsatellite Repeats from Fungal Genomes. HELIX.

[20] Karaoglu H., Lee C.M., Meyer W. (2004). Survey of Simple Sequence Repeats in Completed Fungal Genomes. Mol. Biol. Evol..

[21] Boundy-Mills K.L., Glantschnig E., Roberts I.N., Yurkov A., Casaregola S., Daniel H.M., Groenewald M., Turchetti B. (2016). Yeast Culture Collections in the Twenty-First Century: New Opportunities and Challenges. Yeast.

[22] Mokhtar M.M., Atia M. (2019). SSRome: An Integrated Database and Pipelines for Exploring Microsatellites in all Organisms. Nucleic Acids Res..

[23] Richards K.D., Goddard M.R., Gardner R.C. (2009). A Database of Microsatellite Genotypes for Saccharomyces Cerevisiae. Antonie Leeuwenhoek.

[24] Prakash P.Y., Irinyi L., Halliday C., Chen S., Robert V., Meyer W. (2017). Online Databases for Taxonomy and Identification of Pathogenic Fungi and Proposal for a Cloud-Based Dynamic Data Network Platform. J. Clin. Microbiol..

[25] Avvaru A.K., Saxena S., Sowpati D.T., Mishra R.K. (2017). MSDB: A Comprehensive Database of Simple Sequence Repeats. Genome Biol. Evol..

[26] Beier S., Thiel T., Münch T., Scholz U., Mascher M. (2017). MISA-Web: A Web Server for Microsatellite Prediction. Bioinformatics.

[27] Thiel T., Michalek W., Varshney R.K. (2003). Exploiting EST Databases for the Development of cDNA Derived Microsatellite Markers in Barley (*Hordeum vulgare* L.). Theor. Appl. Genet..

[28] Untergasser A., Cutcutache I., Koressaar T., Ye J., Faircloth B.C., Remm M., Rozen S.G. (2012). Primer3—New Capabilities and Interfaces. Nucleic Acids Res..

[29] Das R., Arora V., Jaiswal S., Iquebal M.A., Angadi U.B., Fatma S., Singh R., Shil S., Rai A., Kumar D. (2018). PolyMorphPredict: Web Server for Rapid Polymorphic SSR Locus Discovery from Whole Genome and Transcriptome Data. Front. Plant Sci..

[30] Cenis J.L. (1992). Rapid Extraction of Fungal DNA for PCR Amplification. Nucleic Acids Res..

[31] Lee S.B., Milgroom M.G., Taylor J.W. (1988). A Rapid, High Yield Mini-Prep Method for Isolation of Total Genomic DNA from Fungi. Fungal Genet Newsl..

[32] White T.J., Bruns T., Lee S.J.W.T., Taylor J., Innis M.A., Gelfand D.H., Sninsky J.J., White T.J. (1990). Amplification and Direct Sequencing of Fungal Ribosomal RNA Genes for Phylogenetics. PCR Protocols: A Guide to Methods and Applications.

[33] Rao S., Sharda S., Oddi V., Nandineni M.R. (2018). The Landscape of Repetitive Elements in the Refined Genome of Chilli Anthracnose Fungus Colletotrichum Truncatum. Front. Microbiol..

[34] Cai G., Leadbetter C.W., Muehlbauer M.F., Molnar T.J., Hillman B.I. (2013). Genome-Wide Microsatellite Identification in the Fungus Anisogramma Anomala Using Illumina Sequencing and Genome Assembly. PLoS ONE.

[35] Kelkar Y.D., Strubczewski N., Hile S.E., Chiaromonte F., Eckert K.A., Makova K.D. (2010). What is a Microsatellite: A Computational and Experimental Definition Based Upon Repeat Mutational Behaviour at A/T and GT/AC Repeats. Genome Biol. Evol..

[36] Longato S., Bonfante P. (1997). Molecular Identification of Mycorrhizal Fungi by Direct Amplification of Microsatellite Regions. Mycol. Res..

[37] Della R.V., Cappuccio I., Fanelli C., Urbanelli S. (2004). Isolation and Characterization of Microsatellite Markers in Two Basidiomycete Species: Pleurotus Eryngii and P. Ferulae. Mol. Ecol. Notes.

[38] Jackson C.J., Barton R.C., Evans E.G. (1999). Species Identification and Strain Differentiation of Dermatophyte Fungi by Analysis of Ribosomal-DNA Intergenic Spacer Regions. J. Clin. Microbiol..

[39] Bidochka M.J., McDonald M.A., Leger R.J.S., Roberts D.W. (1994). Differentiation of Species and Strains of Entomopathogenic Fungi by Random Amplification of Polymorphic DNA (RAPD). Curr. Genet..

[40] Bajinka O., Terzi Y., Ucar F. (2017). The Development of Diagnostics Tools and Techniques in the Isolation and Detection of Fungal Pathogens. J. Infect. Dis. Med..

[41] Coronado-Ruiz C., Avendaño R., Escudero-Leyva E., Conejo-Barboza G., Chaverri P., Havarría M. (2018). Two New Cellulolytic Fungal Species Isolated from a 19th-Century Art Collection. Sci. Rep..

[42] Gobbin D., Pertot I., Gessler C. (2003). Identification of Microsatellite Markers for Plasmopara Viticola and Establishment of High throughput Method for SSR Analysis. Eur. J. Plant Pathol..

[43] Tarakanta J.A., Sharma T.R., Singh N.K. (2005). SSR-Based Detection of Genetic Variability in the Charcoal Root Rot Pathogen Macrophomina Phaseolina. Mycol. Res..

[44] Moges A.D., Admassu B., Belew D., Yesuf M., Njuguna J., Kyalo M., Ghimire S.R. (2016). Development of Microsatellite Markers and Analysis of Genetic Diversity and Population Structure of Colletotrichum Gloeosporioides from Ethiopia. PLoS ONE.

[45] Visser B., Herselman L., Park R.F., Karaoglu H., Bender C.M., Pretorius Z.A. (2011). Characterization of Two New Puccinia Graminis f. sp. Tritici Races Within the Ug99 Lineage in South Africa. Euphytica.

[46] Visser B., Herselman L., Pretorius Z.A. (2009). Genetic Comparison of Ug99 with Selected South African Races of Puccinia Graminis f.sp. Tritici. Mol. Plant Pathol..

[47] Ebrahimi L., Fotuhifar K.B., Nikkhah M.J., Naghavi M.R., Baisakh N. (2016). Population Genetic Structure of Apple Scab (Venturia Inaequalis (Cooke) G. Winter) in Iran. PLoS ONE.

[48] Bailey J., Karaoglu H., Wellings C.R., Park R.F. (2015). PCR-Based Simple Sequence Repeat Markers for Diagnostic Identification of Major Clonal Lineages of Puccinia Striiformis f. sp. Tritici and Related Stripe Rust Pathogens in Australia. Australas. Plant Pathol..

[49] Lees A.K., Wattier R., Shaw D.S., Sullivan L., Williams N.A., Cooke D.E.L. (2006). Novel Microsatellite Markers for the Analysis of Phytophthora Infestans Populations. Plant Pathol..

[50] Martin F.N., Zhang Y., Cooke D.E., Coffey M.D., Grünwald N.J., Fry W.E. (2019). Insights into Evolving Global Populations of Phytophthora Infestans via New Complementary mtDNA Haplotype Markers and Nuclear SSRs. PLoS ONE.

[51] McDonald B.A. (1997). The Population Genetics of Fungi: Tools and Techniques. Phytopathology.

[52] Geistlinger J., Zwanzig J., Heckendorff S., Schellenberg I. (2015). SSR Markers for Trichoderma Virens: Their Evaluation and Application to Identify and Quantify Root-Endophytic Strains. Diversity.

[53] Canfora L., Malusà E., Tkaczuk C., Tartanus M., Łabanowska B.H., Pinzari F. (2016). Development of a Method for Detection and Quantification of B. Brongniartii and B. Bassiana in Soil. Sci. Rep..

[54] Gauthier N., Dalleau-Clouet C., Fargues J., Bon M.C. (2007). Microsatellite Variability in the Entomopathogenic Fungus Paecilomyces Fumosoroseus: Genetic Diversity and Population Structure. Mycologia.

[55] Tõrra T., Cornejo C., Cheenacharoen S., Dal Grande F., Marmor L., Scheidegger C. (2014). Characterization of Fungus-Specific Microsatellite Markers in the Lichen Fungus Usnea Subfloridana (Parmeliaceae). Appl. Plant Sci..

[56] Arias R.S., Ray J.D., Mengistu A., Scheffler B.E. (2011). Discriminating Microsatellites from Macrophomina Phaseolina and Their Potential Association to Biological Functions. Plant Pathol..

[57] Suranska H., Vranova D., Omelkova J. (2016). Isolation, Identification and Characterization of Regional Indigenous Saccharomyces cerevisiae Strains. Braz. J. Microbiol..

[58] Ayoub M.J., Legras J.L., Saliba R., Gaillardin C. (2006). Application of Multi Locus Sequence Typing to the Analysis of the Biodiversity of Indigenous Saccharomyces Cerevisiae Wine Yeasts from Lebanon. J. Appl. Microbiol..

[59] Zacharof M.P. (2017). Grape Winery Waste as Feedstock for Bioconversions: Applying the Biorefinery Concept. Waste Biomass Valorization.

[60] Bart-Delabesse E., Cordonnier C., Bretagne S. (1999). Usefulness of Genotyping with Microsatellite Markers to Investigate Hospital-Acquired Invasive Aspergillosis. J. Hosp. Infect..

[61] Galagan J.E., Henn M.R., Ma L.J., Cuomo C.A., Birren B. (2005). Genomics of the Fungal Kingdom: Insights into Eukaryotic Biology. Genome Res..

